# Professional quality of life amongst nurses in psychiatric observation units

**DOI:** 10.4102/sajpsychiatry.v26i0.1553

**Published:** 2020-08-25

**Authors:** Siyavuya Maila, Penelope D. Martin, Jennifer Chipps

**Affiliations:** 1 School of Nursing, University of the Western Cape, Cape Town, South Africa

**Keywords:** Burnout, Compassion Fatigue, Compassion Satisfaction, Professional Quality of Life, Secondary Traumatic Stress

## Abstract

**Background:**

Professional quality of life amongst nurses in psychiatric observations units may be affected by working conditions such as an overflow of mental health care users (MHCUs), a shortage of nurses, lack of specialised staff and inadequate infrastructure to accommodate MHCUs amongst others.

**Aim:**

The aim of the study was to investigate the professional quality of life amongst nurses in psychiatric observation units.

**Setting:**

The study was conducted in psychiatric observation units in eight hospitals in the Metropole District Health Services in the Western Cape.

**Method:**

A quantitative descriptive survey design using the Professional Quality of Life (ProQoL version 5) questionnaire was conducted with an all-inclusive sample of 175 nurses. The ProQoL has two scales, namely, the compassion satisfaction and the compassion fatigue. Compassion fatigue includes two subscales, burnout and secondary traumatic stress. Ethics to conduct the study was obtained from the Research Ethics Committee at the university and the Department of Health in the Western Cape.

**Results:**

A response rate of 93% (*n* = 163) was obtained. Respondents reported moderate compassion satisfaction. Psychiatric nurse specialists and registered nurses reported lower compassion satisfaction than enrolled nurses and nursing assistants. This came with moderate levels of burnout and high levels of secondary traumatic stress, with enrolled nurses and enrolled nursing assistants reporting lower levels than the other professional groups.

**Conclusion:**

Psychiatric nurse specialists and registered nurses experienced higher burnout and secondary traumatic stress and lower compassion satisfaction than the lower categories of nurses.

## Introduction

South Africa has a high prevalence of mental health problems with 30.3% reported common mental health problems and a 47.5% projected risk of mental disorders during a lifetime.^1^ This is concomitant with a large mental health treatment gap of 92%.^2^ *The Mental Health Care Act No. 17 of 2002* aims to bridge this gap by integrating mental healthcare services into primary healthcare, specifically with the creation of 72-h observation units (psychiatric observation units) in selected general hospitals for Mental Health Care Users (MHCUs) requiring involuntary admission. The creation of these 72-h observation units have contributed to high patient turnover and overflow, longer length of stays and inadequate or limited financial, infrastructure and human resources.^3^ These observation units are often short staffed and manned by non-specialist psychiatric nurses.^4^ In KwaZulu-Natal, almost 70% of hospitals did not have enough-skilled nursing and medical staff to render required mental health services.^3^

Compassion fatigue impacts on compassion satisfaction (the fulfilment attained from helping and caring for others).^5,6^ Compassion fatigue includes experiences of burnout (feeling drained and emotionally worn-out)^7,8^ and secondary traumatic stress (emotional strain associated with the exposure to stressful, traumatic events and danger at work).^5,6^

Professional quality of life in nurses has been investigated in general and mental health establishments globally,^9,10,11,12,13,14^ and in general health establishments in South Africa.^15,16,17,18^ These studies have found that nurses are predisposed to burnout and secondary traumatic stress more than any other healthcare providers because of long hours in health facilities, administrative workload, supervision and staffing challenges.^7,19^ In mental healthcare establishments as in general health, professional quality of life for nurses is an important aspect of career satisfaction and retention.^14,20,21^

Burnout amongst nurses working in psychiatric hospitals are common^22^ and has been attributed to the scarcity of essential requirements in hospitals such as skilled staff, equipment, budgets,^23,24^ high clinical and administrative workloads of nurses,^3,7,25^ exposure to chronic MHCUs^26^ and the prolonged stay of MHCUs^3,25^ and the prolonged exposure to direct and indirect trauma in the units.^27^ In addition, the nature of acute admissions to 72-h observation units may expose nurses to aggression related to acute illness,^13^ especially during involuntary admissions.^20^

In South Africa, there are four nursing categories^28^: working in psychiatric observation units; enrolled nursing assistants and enrolled nurses who have no formal education or training in psychiatric nursing apart from in-service training; registered nurses who have completed a 4-year diploma or degree in nursing (general, community and psychiatry) and midwifery; and psychiatric nurse specialists who have an advanced qualification in psychiatric nursing science.^28^ Internationally, registered nurses were reported to have lower secondary traumatic stress than nursing assistants,^21^ although registered nurses working in child and adolescent psychiatry were reported to have higher burnout than nursing assistants.^14^ Patient’s needs, type of illnesses and symptom and the administrative roles in the unit and staff shortages were all reported as stressors related to burnout by registered nurses, whilst enrolled nurses and enrolled nursing assistants reported shortage of staff, challenges involved with patient care and lack of acknowledgement for their hard work.^29^

To date, no study has been carried out in South Africa to assess the professional quality of life of these nurse categories, and no study has specifically assessed the specific impact of psychiatric observation units on these nurse categories. This study aimed to investigate the professional quality of life amongst nurses in psychiatric observations units in the Metropole District Health Services in the Western Cape, South Africa.

## Research methods and design

### Study design

A survey was conducted with all nurse categories.

### Setting

The setting of the study was 16 psychiatric observation units in eight hospitals in the Metropole District Health Services in the Western Cape, South Africa.

### Study population and sampling strategy

The population for this study was 175 nurses (14 psychiatric nurse specialists, 59 registered nurses, 24 enrolled nurses and 78 enrolled nursing assistants), all providing direct nursing care to MHCUs. An all-inclusive sampling strategy was used.

### Instrument

The 30-itemed, 5-point Likert scale, Professional Quality of Life 5 instrument (ProQoL 5) was used (with permission).^6^ The ProQoL 5 is a validated instrument which aims to the assess respondents’ feelings or experiences of compassion satisfaction and compassion fatigue in the last 30 days. The ProQoL has two scales, compassion satisfaction and compassion fatigue. Compassion fatigue has two subscales, namely burnout and secondary traumatic stress.^6^ The ProQoL 5 has acceptable reliability and validity with Cronbach’s alpha scale reliability for the subscales (for compassion satisfaction, α = 0.88; for burnout, α = 0.75, and for secondary traumatic stress, α = 0.81) and has good construct validity in more than 200 articles which have been published.^6^

### Data collection

The researcher visited the eight hospitals to obtain permission to gain access for data collection. Information sheets were handed to all potential respondents to explain the study and to obtain their consent. The data were collected from August 2018 to October 2018.

### Data analysis

Data were analysed using SPSS version 25 (SPSS Inc., Chicago, IL, USA). Demographic characteristics and the ProQoL individual items were analysed using descriptive statistics. The scores for the sub-scales were calculated using the raw scores and using the ProQoL manual’s cut points, categories for the low, middle and high scores for compassion satisfaction (score > 42 = high, between 32 and 42 = moderate and < 32 = low)^5,6^; burnout (score > 27 = high, between 18 and 27 = moderate, and < 18 = low)^5,6^; and secondary traumatic stress (score > 17 = high, between 8 and 17 = moderate, and < 8 = low) were coded.^5,6^ Differences between the categories of nurses were measured using 95% confidence interval (CI), chi-square tests (*X*^*2*^) and independent samples Kruskal–Wallis tests (*K*). Significance was set at *p* < 0.05.

### Ethical consideration

Ethical clearance was obtained from the Biomedical Research Council in the University of the Western Cape (ethics reference number: BM18/5/21) and Western Cape Government Health, reference number: WC-201807_024.

## Results

Of the 175 nurses who participated in the study, 163 submitted completed the questionnaires (93% response rate). Two scales showed adequate internal consistency, with Cronbach’s α for compassion satisfaction α = 0.763 and secondary traumatic stress α = 0.741. The internal consistency for the burnout scale was lower at α = 0.590.

### Demographics

Of the 163 respondents, 69.3% (113) were female respondents and 63.2% (103) of the respondents reported being single. The average age of the respondents was 37.5 (± 9.4) years. Nearly half of the respondents were enrolled nursing assistants (68, 41.9%), followed by registered nurses (60, 36.8%), enrolled nurses (21, 12.9%) and psychiatric specialist nurses (14, 8.6%) (Table 1). The average years of experience working as nurses were 7.5 (± 8.4) (median 4 years, ranging from 1 to 35 years). There were significant differences amongst the respondents in the nurse categories in terms of gender, with higher proportions of male psychiatric nurse specialist respondents compared with registered nurses, enrolled nurses and enrolled nursing assistant respondents 10 (71.4%) versus 18 (30%) versus 9 (42.9%) versus 13 (19.1%), respectively (χ^2^ = 16.6, *p* = 0.001).

**TABLE 1:** Demographics of respondents (*n* = 163).

Items	All (*n* = 163)					Psychiatric nurse specialists *n* = 14 (8.6%)					Registered nurses *n* = 60 (36.8%)					Enrolled nurses *n* = 21 (12.9%)					Enrolled nursing assistants *n* = 68 (41.9%)					Test	*p*
	*n*	%	Mean	SD	Median	*n*	%	Mean	SD	Median	*n*	%	Mean	SD	Median	*n*	%	Mean	SD	Median	*n*	%	Mean	SD	Median		
**Gender**																										*X^2^ =* 16.6	0.001*
Male	50	30.7	-	-	-	10	71.4	-	-	-	18	30	-	-	-	9	42.9	-	-	-	13	19.1	-	-	-		
Female	113	69.3	-	-	-	4	28.6	-	-	-	42	70	-	-	-	12	57.1	-	-	-	55	80.9	-	-	-		
**Marital status**																										*X*^2^ = 12.2	0.202
Single	103	63.2	-	-	-	7	50	-	-	-	36	60	-	-	-	16	76.2	-	-	-	44	64.7	-	-	-		
Married	48	29.4	-	-	-	7	50	-	-	-	20	33.3	-	-	-	3	14.3	-	-	-	18	26.5	-	-	-		
Divorced	7	4.3	-	-	-	0	0	-	-	-	2	3.3	-	-	-	0	0.0	-	-	-	5	7.4	-	-	-		
Widowed	5	3.1	-		-	0	0	-	-	-	2	3.3	-	-	-	2	9.5	-	-	-	1	1.5	-	-	-		
**Age, (years)**	-	-	37.5	± 9.4	-	-	-	41.8	± 8.1	-	-	-	36.1	± 10.9	-	-	-	34.6	± 7.3	-	-	-	38.6	± 8.4		*K* = 9.1	0.020*
**Experience (years)**	-	-	7.5	± 8.4	4.0	-	-	11.7	± 8.2	9.5	-	-	8.7	± 10.2	5.0	-	-	4.7	± 5.8	4.0	-	-	6.5	± 7.0	4.0	*K* = 10.9	0.010*

SD, standard deviation; χ^2^, chi-square test or Fisher’s exact test where relevant; *K*, independent samples Kruskal–Wallis tests.

* , Significant at *p* < 0.05.

Enrolled nurse respondents were significantly younger than psychiatric nurse specialist, registered nurses and enrolled nursing assistant responders (34.6 vs. 41.8 vs. 36.1 vs. 38.6 years, respectively (*K* = 9.1, *p* = 0.020). Enrolled nurse respondents also reported significantly less years of experience than psychiatric nurse specialist, registered nurse and enrolled nursing assistant respondents’ (4.0 vs. 9.5 vs. 5.0 vs. 4.0 median) years of experience, respectively *K*^2^ = 14.2, *p* = 0.027) (Table 1).

### Professional quality of life amongst nurses in psychiatric observations units

Overall, the respondents reported moderate compassion satisfaction (41.6 [CI 95% 40.7–42.5]), moderate burnout (24.6 [CI 95% 23.7–25.5]) and high secondary traumatic stress (27.36 [CI 95% 26.2–28.4]) (Table 2).

**TABLE 2:** Compassion satisfaction, burnout and secondary traumatic stress scores by nurse categories.

Subscales	Total *N* = 163 [CI 95%]			Psychiatric nurse specialists *n* = 14			Registered nurses *n* = 60			Enrolled nurses *n* = 21			Enrolled nursing assistants *n* = 68 (41.9%)			Test	*p*
	Mean	SD	CI 95%	Mean	SD	CI 95%	Mean	SD	CI 95%	Mean	SD	CI 95%	Mean	SD	CI 95%		
Compassion satisfaction	41.6	± 0.4	40.7–42.5	40.2	± 1.9	35.9–44.5	40	± 0.6	38.6–41.4	42.4	± 0.8	40.6–44.2	43	± 0.6	41.7–44.3	*K* = 12.2	0.007*
Burnout	24.6	± 0.4	23.7–25.5	26.9	± 1.6	23.4–30.4	25.4	± 0.7	23.9–26.8	21.8	± 1.1	19.4–24.2	24.3	± 0.6	23.0–25.7	*K* = 8.3	0.040*
Secondary traumatic stress	27.36	± 0.5	26.2–28.4	27.6	± 6.1	25–30.2	28.3	± 0.9	26.4–30.2	25.7	± 1.4	22.7–28.8	26.9	± 0.9	25.0–28.7	*K* = 2.3	0.496

SD, standard deviation; CI, confidence interval; *K*, independent samples Kruskal–Wallis tests.

* , Significance set at *p* ≤ 0.05.

### Compassion satisfaction

The respondents reported moderate compassion satisfaction (41.6 [CI 95% 40.7–42.5]), with the highest rating for *I am proud of what I can do to help* (4.4 [CI 95% 4.2–4.5]) and the lowest for *I feel invigorated after working with those I nurse* (3.3 [CI 95% 3.1–3.3]) (Table 3). These differences were because of enrolled nursing assistants and enrolled nurse respondents rating the following statements significantly higher: *I am happy I chose to do this work* (4.7 vs. 4.5, *K* = 25.1, *p* < 0.001); and *I am pleased with how I am able to keep up with nursing techniques and protocols* (4.3 vs. 4.2, *K* = 8.6, *p* = 0.034) (Table 3).

**TABLE 3:** Compassion satisfaction by nurse category.

Compassion satisfaction statements	All *N* = 163			Psychiatric nurse specialists *n* = 14		Registered nurses *n* = 60		Enrolled nurses *n* = 21		Enrolled nursing assistants *n* = 68		Test	*p*
	Mean	SD	CI 95%	Mean	SD	Mean	SD	Mean	SD	Mean	SD		
I am proud of what I can do to help	4.4	± 0.9	4.2–45	4.2	± 1.1	4.3	± 0.8	4.5	± 0.7	4.4	± 0.9	*K* = 1.2	0.730
I believe I can make a difference through my work	4.3	± 0.8	4.2–4.5	4	± 1	4.2	± 0.9	4.3	± 0.9	4.5	± 0.7	*K* = 7.5	0.057
I am happy I chose to do this work	4.3	± 0.9	4.1–4.4	3.7	± 1.2	3.9	± 1	4.5	± 0.8	4.7	± 0.6	*K* = 25	< 0.001*
I have happy thoughts and feelings about of those I nurse and how I could help them	4.2	± 0.8	4–4.2	4.1	± 0.7	4.1	± 0.8	4.4	± 0.7	4.2	± 0.9	*K* = 2	0.570
I have thoughts that I am ‘successful’ as a nurse	4.0	± 1	3.8–4.1	3.8	± 1.0	3.8	± 1	4.1	± 1	4.1	± 1.1	*K* = 3	0.316
I like my work as a nurse	4.5	± 0.8	4.4–4.6	4.4	± 0.9	4.3	± 0.9	4.9	± 0.3	4.7	± 0.6	*K* = 14	0.002*
I feel satisfied from being able to nurse people	4.3	± 0.8	4.2–4.5	4	± 1	4.4	± 0.7	4.1	± 0.8	4.3	± 0.9	*K* = 3	0.382
I am pleased with how I am able to keep up with nursing techniques and protocols	4.1	± 0.9	3.9–4.2	4.2	± 0.9	3.8	± 0.9	4.2	± 1	4.3	± 0.9	*K* = 8	0.034*
My work makes me feel satisfied	3.9	± 1.1	3.7–4.1	4	± 1.2	3.7	± 0.9	3.9	± 1.1	4.1	± 1.1	*K* = 6.4	0.090
I feel invigorated after working with those I nurse	3.3	± 1.2	3.1–3.3	3.4	± 1.2	3.1	± 1	3.3	± 1.4	3.5	± 1.2	*K* = 3.6	0.300

SD, standard deviation; CI, confidence interval; *K*, independent samples Kruskal–Wallis tests.

* , Significance at *p* < 0.05.

### Burnout

Overall, the respondents reported moderate burnout scores (24.6 [CI 95% 23.7–25.5]), with the highest rating for *I feel overwhelmed because my case workload seems endless* (3.2 [CI 95% 3–3.4]) and the lowest rating for *I am a very caring person* (1.6 [CI 95% 1.5–1.8]) (Table 4). *I feel ‘bogged’ down by the system* (3.7 vs. 3.3 vs. 2.6 and 3, χ^2^ = 8.3 *p* = 0.039*), *I feel trapped in my job as a nurse* (3.0 vs. 2.3 vs. 1.7 vs. 1.9, χ^2^ = 10.6 *p* = 0.014*) and *I feel overwhelmed because in my case work load seems endless* (4 vs. 3.3 vs. 2.6 vs. 3.2, *K* = 9, *p* = 0.028*) were rated significantly higher by the psychiatric nurse specialist than the registered nurse, enrolled nurse and enrolled nursing assistant respondents (Table 4).

**TABLE 4:** Burnout by nurse category.

Burnout statements	All *n* = 163			Psychiatric nurse specialist *n* = 14		Registered nurse *n* = 60		Enrolled nurse *n* = 21		Enrolled nursing assistant *n* = 68		Test	*p*
	Mean	SD	CI 95%	Mean	SD	Mean	SD	Mean	SD	Mean	SD		
I feel overwhelmed because my case workload seems endless	3.2	± 1.2	3.0–3.4	4.0	± 0.8	3.3	± 1.1	2.6	± 1.6	3.2	± 1.2	*K* = 9.0	0.028*
I feel worn out because of my work as a nurse	3.2	± 1.3	3.0–3.4	3.7	± 1.3	3.3	± 1.1	2.6	± 1.4	3.3	± 1.3	*K* = 6.5	0.089
I feel ‘bogged’ down by the system	3.1	± 1.2	2.9–3.3	3.7	± 1.1	3.3	± 1.3	2.6	± 1.2	3.0	± 1.2	*K* = 8.3	0.039*
I have belief system that sustain me	2.6	± 1.3	2.3–2.8	2.0	± 1.2	2.5	± 1.4	2.8	± 1.2	2.7	± 1.3	*K* = 4.2	0.237
I am the person I always wanted to be	2.2	1.3	2.0–2.4	2.0	± 1.2	2.5	± 1.3	2.5	± 1.4	1.9	± 1.3	*K* = 10.2	0.016*
I am happy	2.0	± 1.0	1.9–2.2	2.2	0.7	2.0	± 0.9	1.7	± 0.9	2.1	± 1.2	*K* = 4.1	0.247
I feel trapped in my job as a nurse	2.1	± 1.3	1.9–2.4	3.0	± 1.4	2.3	± 1.3	1.7	± 1.2	1.9	± 1.3	*K* = 10.6	0.014*
I feel connected to others	2.1	± 1.0	1.9–2.2	2.0	± 1.0	2.1	± 1.0	1.8	± 0.9	2.1	± 1.2	*K* = 1.5	0.672
I am not as productive at work because I am losing sleep over traumatic experiences of a person I nursed	2.0	± 1.2	1.8–2.2	2.2	± 0.8	1.9	± 1.2	1.7	± 1.0	2.1	± 1.3	*K* = 3.4	0.322
I am a very caring person	1.6	± 1.1	1.5–1.8	1.7	± 0.9	1.7	± 1.0	1.5	± 1.0	1.7	± 1.0	*K* = 3.1	0.374

SD, standard deviation; CI, confidence interval; *K*, independent samples Kruskal–Wallis tests.

* , Significance set at *p* ≤ 0.05.

### Secondary traumatic stress

The respondents reported high secondary traumatic stress scores (27.36 [CI 95% 26.2–28.4]), with the highest rating for *I am preoccupied with more than one person I nurse* (3.5 [CI 95% 3.3–3.7]) and the lowest for *I feel depressed because of traumatic experiences of the people I nursed* (2.4 [CI 95% 2.2–2.5]) (Table 5). There were no overall significant differences between respondents nurse categories (Table 2). *I think that I might have been affected by the traumatic stress of those I nurse* was rated higher by psychiatric nurse specialist respondents compared with registered nurses and enrolled nursing assistant respondents (2.7 vs. 2.4 vs. 1.7 vs. 2.2 vs. *K* = 6.8 *p* = 0.077), although not significant (Table 5).

**TABLE 5:** Secondary traumatic stress statements.

Secondary traumatic stress statements	All *N* = 163			Psychiatric nurse specialists *n* = 14		Registered nurses *n* = 60		Enrolled nurses *n* = 21		Enrolled nursing assistants *n* = 68		Test	*p*
	Mean	SD	CI 95%	Mean	SD	Mean	SD	Mean	SD	Mean	SD		
I am preoccupied with more than one person I nurse	3.5	± 1.3	3.3–3.7	4.1	± 0.8	3.6	± 1.2	3.3	± 1.3	3.4	± 1.4	*K* = 3.3	0.343
I jump or am startled by unexpected sounds	2.9	± 1.2	2.7–3.1	2.5	± 1.0	3.2	± 1.1	2.7	± 1.2	2.8	± 1.3	*K* = 5.9	0.113
I find it difficult to separate personal life from my life as a nurse	2.9	± 1.4	2.2–2.7	2.5	± 1.3	2.5	± 1.4	2.1	± 1.4	2.5	± 1.4	*K* = 2.0	0.572
Because of my work, I have felt ‘on edge’ on various things	2.8	± 1.2	2.6–3.3	3.3	± 1.1	2.9	± 1.1	2.6	± 1.1	2.6	± 1.2	*K* = 4.4	0.216
I think that I might have been affected by the traumatic stress of those I nurse	2.2	± 1.2	2.1–2.4	2.7	± 0.9	2.4	± 1.2	1.7	± 0.8	2.2	± 1.2	*K* = 6.8	0.077
I can’t recall important parts of my work with trauma victims	2.9	± 1.2	2.2–2.6	2.3	± 0.6	2.7	± 1.1	3.0	± 1.5	3.1	± 1.2	*K* = 6.2	0.102
As a result of my helping, I have intrusive frightening thoughts	2.7	± 1.5	2.2–2.6	2.5	± 1.3	2.9	± 1.4	2.8	± 1.6	2.6	± 1.5	*K* = 1.3	0.727
I avoid certain activities or situations because they remind of frightening experiences of the people I nurse	2.5	± 1.3	2.2–2.6	2.5	± 0.9	2.6	± 1.3	2.6	± 1.4	2.5	± 1.3	*K* = 0.3	0.954
I feel as if I am experiencing the trauma of someone I have nursed	2.5	± 1.2	2.2–2.6	2.3	± 0.8	2.6	± 1.3	2.5	± 1.2	2.5	± 1.3	*K* = 0.59	0.898
I feel depressed because of traumatic experience of the people I nurse	2.4	± 1.2	2.2–2.5	2.5	± 0.8	2.6	± 1.3	2.1	± 1.1	2.2	± 1.1	*K* = 3.0	0.391

SD, standard deviation; CI, confidence interval; *K*, independent samples Kruskal–Wallis tests.

* , Significance set at *p* ≤ 0.05.

## Discussion

Overall, all the respondents reported moderate compassion satisfaction, moderate burnout but high secondary traumatic stress. Enrolled nursing assistant respondents had significantly higher compassion satisfaction scores than the other nursing categories, and these respondents reported that they were happy working with MHCUs and felt that they were able to keep abreast of nursing techniques in rendering care for MHCUs. The higher compassion satisfaction in enrolled nursing assistants may be because of the significantly shorter years of experience (median 4.0 years) compared with the psychiatric nursing specialists (median 9.5 years). Although no studies have been carried out on professional quality of life in these different categories of nurses working in mental health establishments, studies in South Africa in a maternity setting in contrast found that registered nurses reported higher compassion satisfaction than enrolled nurses.^15^

In this study, all respondents reported high secondary traumatic stress. The high levels of secondary stress and burnout may be because of the nature of mental healthcare which includes being exposed to the trauma associated with mental illness,^4^ frequent involuntarily admissions of MHCUs^4^ and exposure to aggressive behaviour from MHCUs.^30^ Other studies have also reported the high secondary traumatic stress related to being assaulted by MHCUs.^13,31,32^

Psychiatric nurse specialist respondents reported higher burnout than the other nursing categories and reported feelings of being bogged down, trapped, overwhelmed and preoccupied with the MHCUs. The higher ratings of burnout by specialist psychiatric nurse respondents specifically may be related to the expectations placed on specialist psychiatric nurses who are expected to render specialist mental healthcare, treatment and rehabilitation commensurate with their education and training.^3,4^ One of the roles of psychiatric nurse specialists is to render psychotherapeutic interventions to MHCUs and the therapeutic process of listening to traumatic experiences and employing empathy may predispose them to secondary trauma.^33,34,35^ Similar findings were reported in Northern England where higher secondary traumatic stress and burnout were found in registered mental health nurses compared with healthcare assistants (similar to enrolled nurse assistants).^14^ These reports in mental health are similar to trends in other health settings such as maternity where registered nurses had higher ratings of compassion fatigue than enrolled and nursing assistants.^15^

A second factor which may contribute to differences in burnout in the nurse categories may be because of differences in professional educational preparation in these categories. A lack of formal psychiatric education and training has been identified as a predisposition to emotional and physical exhaustion,^36^ which can lead to secondary traumatic stress and/or burnout. In contrast, a study in Greece reported higher secondary traumatic stress amongst nurse assistants than registered nurses which they attributed to nurse assistants having more direct contact with MHCUs and less psychiatric training.^21^

Lastly, working conditions such as poor infrastructure and high workload experienced in psychiatric observation units are also thought to contribute to secondary traumatic stress. The psychiatric nurse specialists and registered nurses in these units are expected to lead and be accountable for the management of acutely disturbed MHCUs, and the support from a multi-disciplinary team may be limited,^4^ as specialised staff such as psychiatrists, psychologists, social workers and occupational therapists are scarce.^3,4^

## Conclusion

Psychiatric nurse specialists and registered nurses experienced higher burnout and secondary traumatic stress and lower compassion satisfaction than the other categories of nurses. Professional quality of life for nurses is an important aspect of career satisfaction and retention,^37^ and the on-going investigation of professional quality of life of all nurse categories working in mental health settings is essential.^38^ This is especially important for specialist psychiatric nurses to ensure job satisfaction and to retain an experienced workforce for mental healthcare.
